# NKG2D^+^ IFN-γ^+^ CD8^+^ T Cells Are Responsible for Palladium Allergy

**DOI:** 10.1371/journal.pone.0086810

**Published:** 2014-02-12

**Authors:** Mitsuko Kawano, Masafumi Nakayama, Yusuke Aoshima, Kyohei Nakamura, Mizuho Ono, Tadashi Nishiya, Syou Nakamura, Yuri Takeda, Akira Dobashi, Akiko Takahashi, Misato Endo, Akiyo Ito, Kyosuke Ueda, Naoki Sato, Shigehito Higuchi, Takeru Kondo, Suguru Hashimoto, Masamichi Watanabe, Makoto Watanabe, Tetsu Takahashi, Keiichi Sasaki, Masanori Nakamura, Takehiko Sasazuki, Takayuki Narushima, Ryuji Suzuki, Kouetsu Ogasawara

**Affiliations:** 1 Department of Immunobiology, Institute of Development, Aging and Cancer, Tohoku University, Aoba-ku, Sendai, Miyagi, Japan; 2 Department of Materials Processing, Graduate School of Engineering, Tohoku University, Aramakiaza, Aoba-ku, Sendai, Miyagi, Japan; 3 Graduate School of Medicine, Dentistry and Pharmaceutical Sciences, Okayama University, Kita-ku, Okayama, Japan; 4 Graduate School of Dentistry, Tohoku University, Aoba-ku, Sendai, Miyagi, Japan; 5 Graduate School of Dentistry, Showa University, Shinagawa-ku, Tokyo, Japan; 6 Institute for Advanced Study, Kyushu University, Higashi-ku, Fukuoka, Japan; 7 Department of Rheumatology and Clinical Immunology, Clinical Research Center for Allergy and Rheumatology, Sagamihara National Hospital, National Hospital Organization, Minami-ku, Sagamihara, Kanagawa, Japan; INSERM- CNRS- Univ. Méditerranée, France

## Abstract

Nickel, cobalt, and chromium are well known to be causal agents of allergic contact dermatitis. Palladium (Pd) can also cause allergic disease and exposure results from wide use of this metal in dental restorations and jewelry. Metal allergy is categorized as a delayed-type hypersensitivity, and metal-responsive T cell clones have been isolated from allergic patients. However, compared to nickel, little is known about the pathology of allergic disease mediated by Pd, and pathogenic T cells are poorly understood. To identify the pathogenic T cells that are responsible for onset of Pd allergy, we enriched metal-responsive lymphocytes by sequential adoptive transfer of involved lymph node cells. Here we show that sequential adoptive transfer gradually increased the incidence and the intensity of Pd allergy, and CD8^+^ T cells are responsible for the disease as CD8^+^ T cell-depleted mice and β2-microglobulin-deficient mice did not develop Pd allergy. In addition, we found that draining lymph node cells skewed toward CD8^+^ T cells in response to Pd challenge in 8th adoptive transferred recipient mice. The CD8^+^ T cells expressed NKG2D, a costimulatory molecule involved in the production of IFN-γ. NKG2D ligand was also induced in Pd-injected tissues. Furthermore, both NKG2D ligand-transgenic mice, where NKG2D is downmodulated, and IFN-γ-deficient mice showed impaired Pd allergy. Taken together, these results indicate that IFN-γ-producing NKG2D^+^ CD8^+^ T cells are responsible for Pd allergy and suggest that NKG2D is a potential therapeutic target for treatment of metal allergy.

## Introduction

Metal allergy is thought to be caused by the release of ions from metal materials [1], [2]. Wide use of metal in jewelry, coins, surgical instruments, and dental restorations may be responsible for recent increases in allergy incidence [3]. Furthermore, the occurence of allergic reactions to dental materials is poorly understood [2]. In addition to nickel, cobalt and chromium, it has been reported that palladium (Pd) also causes allergic disease [2]. The incidence of patients sensitized to Pd has increased in recent years because Pd has more frequently been used in dental restorations [4], [5]. However, compared to nickel, little is known about the pathology of allergic disease mediated by Pd.

Metal allergic disease is categorized as a delayed-type hypersensitivity (DTH), which is developed more than 24 hours after exposure to the causal metal. The hallmark of DTH is the recruitment of lymphocytes and inflammatory cells, including T cells and granulocytes, to the site of allergic inflammation [6], [7]. T cells are known to be involved in the development of metal allergy [8]–[10]. Further, since metal ions are thought to function as haptens, T cell-mediated responses likely contribute to allergic diseases [1], [11]. However, the involvement of pathogenic T cells in the development of metal allergy has not yet been explored using animal models.

T cell clones, both CD4^+^ and CD8^+^, have been established from peripheral blood mononuclear cells (PBMCs) of patients with metal allergy and their responsiveness to the causal metal has been analyzed [8]–[10]. Metal ions induced proliferation of the T cells in vitro [9], [12], [13], and some of the T cell clones produced IFN-γ or IL-4 after metal stimulation in vitro [14]–[17] while some produced both T helper (Th) 1- and Th2- type cytokines [1]. However, the subset of pathogenic T cells involved in the development of metal allergy and their cytokine profiles remain controversial.

For optimal activation, T cells must be stimulated through their T cell receptors (TCRs) and a costimulatory receptor. CD28, inducible costimulator (ICOS), and cytolytic T lymphocyte-associated antigen (CTLA) -4 are all involved in the DTH reaction [18]–[21]. NKG2D is a costimulatory receptor expressed on NK cells, CD8^+^ T cells, NKT cells, and γδ T cells [22]–[24]. Furthermore, NKG2D is expressed on activated/memory mouse CD8^+^ T cells, but not on naïve mouse CD8^+^ T cells and CD4^+^ T cells [23]. In human rheumatoid arthritis, NKG2D^+^ CD4^+^ T cells are present in inflammatory tissues [25]. The ligands of NKG2D are known to be *stress-inducible* molecules, which are expressed in inflamed tissues and by transformed cells. In mice, RAE-1 family proteins have been identified as high affinity NKG2D ligands [22]. We have previously demonstrated that pathogenic CD8^+^ T cells express NKG2D, and that this costimulatory molecule is crucial for the development of inflammatory disease [26]; however, costimulatory and effector molecules expressed on pathogenic T cells for metal allergy have not been identified. In this study, we examined whether CD8^+^ T cells function as pathogenic T cells in Pd allergy in animal models, and we investigated whether NKG2D contributes to the development of Pd allergy.

## Materials and Methods

### Ethics statement

Mice were maintained under specific pathogen-free conditions, and all procedures were performed according to the protocols approved by the Institutional Committee for Use and Care of Laboratory Animals of Tohoku University, which was granted by Tohoku University Ethics Review Board (No. 2012AcA-069) and the Guide for Care and Use of Laboratory Animals published by the U.S. National Institutes of Health (NIH publication 85-23, revised 1996). All surgery was performed under anesthesia by isoflurane. For collection of tissue samples, mice were sacrificed by cervical dislocation. All efforts were made to minimize suffering.

### Mice

C57BL/6 mice, BALB/c mice, and BALB/c nu-nu (nude) mice were obtained from CLEA Japan (Tokyo, Japan). C57BL/6 mice deficient in β2-microglobulin (B2m), IFN-γ, or perforin were obtained from the Jackson Laboratory (Bar Harbor, ME). MHC class II (I-A^b^)-deficient mice [27] were kindly provided by D. Mathis, Harvard Medical School, MA. *Raet1e* transgenic mice were generated as described [28]. These mice were maintained under specific pathogen-free conditions, and used according to the guidelines of the institutional animal care and use committee established at Tohoku University.

### Antibodies and reagents

Rat anti-mouse NKG2D monoclonal antibody (mAb) (CX5) was prepared as described previously [29]. Other antibodies were purchased from BioLegend (San Diego, CA, USA), BD Biosciences (San Jose, CA, USA), eBioscience (San Diego, CA, USA), Santa Cruz Biotechnology (Santa Cruz, CA, USA), or Jackson Immunoresearch Laboratories Inc. (West Grove, PA, USA). PdCl_2_ and NiCl_2_ were purchased from Wako Pure Chemical Industries, Ltd. (Osaka, Japan). 1-fluoro-2,4-dinitrobenzene (DNFB) was purchased from SIGMA Aldrich (St Louis, MO, USA).

### Induction of Pd allergy

Pd allergy was induced in mice as described previously [30]. The experimental design is depicted in Fig. S1A and B. In brief, mice were sensitized by i.p. injection of 250 µl of 10 mM PdCl_2_ with 10 µg/ml lipopolysaccharide (LPS) (SIGMA) in PBS or by applying 50 µl of 0.5% DNFB in AOO (aceton∶olive oil = 4∶1) to the shaved abdominal skin. As a control, mice were administered vehicle only (PBS for Pd plus LPS or AOO for DNFB). Ten days later, these mice were challenged with intradermal (i.d.) injection of 20 µl of 0.5 mM PdCl_2_ in PBS or with application of 20 µl of 0.2% DNFB in AOO into ear auricles under anesthesia. Challenge dose and administration route of DNFB was set at 0.2% according to previous studies [31]. Ear thickness was measured before the challenge, and at 24, 48, and 72 hours after challenge using a Peacock dial thickness gauge (Ozaki MFG Co. Ltd., Tokyo, Japan). The difference in ear thickness before and after the challenge was calculated.

### Sequential adoptive transfer model of Pd allergy

The experimental design is depicted in Fig. S1C. Pd allergy was induced in BALB/c mice as described above. Ten days after Pd allergy induction, donor mice were sacrificed, and the submaxillary lymph node cells (Pd-SLN) were isolated. Single cell suspensions of SLNs were prepared by conventional techniques. These cells were adoptively transferred i.v. (1×10^6^ cells/mouse) into naïve BALB/c nude mice. As a negative control, SLN cells were isolated from naïve BALB/c mice (naïve-SLN). Seven days after the adoptive transfer, recipient mice were challenged with Pd and the difference in ear thickness was calculated as described above. A minimum of 7 days after the challenge, Pd-SLN cells were prepared (2nd Pd-SLN cells) and then adoptively transferred into a third round of naïve nude mice to obtain 3rd Pd-SLN cells, which were then challenged with Pd 7 days post T cell transfer. The adoptive transfer was repeated 8 times to obtain 8th Pd-SLN cells.

### Histological analysis

Twenty-four hours after Pd challenge, ear auricles were obtained and embedded into OCT compound, and then immediately frozen and kept at −80°C. The frozen tissues were sliced into 10 µm sections using a cryostat (Leica Microsystems, Wetzlar, Germany). Immunohistochemical staining was performed by conventional techniques. In brief, acetone-fixed sections were washed twice in PBS, after which they were blocked with DAKO Protein Block (DAKO North America Inc., Carpinteria, CA, USA). Sections were then stained with goat anti-mouse CD3ε (Santa Cruz Biotechnology) or normal goat serum (as control) (Jackson Immunoresearch Laboratories Inc.). After washing twice with TBST, intrinsic peroxidase was quenched by 3% H_2_O_2_ in methanol and then sections were soaked in distilled water and washed twice more in TBST. The sections were incubated with horseradish peroxidase (HRP) -labeled rabbit anti-goat IgG (Nichirei, Tokyo, Japan). After washing twice, samples were incubated with 3,3′-diaminobenzidine (DAB) chromogen (Vector Laboratories Inc., Burlingame, CA, USA), and then sections counter-stained with hematoxylin. The DAB signals were detected using an Olympus IX81 microscope, an Olympus DP71 CCD camera (Olympus, Tokyo, Japan) and LuminaVision software (Mitani Corporation, Fukui, Japan).

For image cytometry, immunohistochemical-stained images were analyzed using Histoquest software (TissueGnostics, Vienna, Austria). Briefly, nucleated cells were detected by a dissection algorithm in the hematoxylin channel; then, DAB-positive cells were detected by signals in the nucleated cells. DAB intensity was plotted against hematoxylin intensity to create the scattergrams. The cut-off threshold was uniform for all images.

### Flow cytometric analysis

SLN cells were pretreated with anti-CD16 + CD32 mAb (2.4G2) (BD Biosciences) to block Fc receptors, and then stained with the combination of fluorescein isothiocyanate (FITC) -labeled anti-CD4 (GK1.5), allophycocyanin (APC) -labeled anti-CD8 (53-6.7), and phycoerythrin (PE) -labeled anti-NKG2D (CX5) mAb or the combination of FITC-labeled anti-CD4 (GK1.5), APC-labeled anti-TCR β (H57-597) and PE-labeled anti-CD8 mAb (53-6.7). For an isotype-matched control of anti-NKG2D mAb, PE-labeled rat IgG_1_ was used. These cells were washed twice with PBS and stained with propidium iodide (Sigma Aldrich). Expression of each cell surface antigen was analyzed on a FACSCanto II with CellQuest software (BD Biosciences).

### Cytokine production

Fifteen hours after challenge with 0.5 mM PdCl_2_ or 0.2% DNFB in ear auricles, mice were sacrificed and then SLN cells were isolated. These cells were stimulated with 20 ng/ml phorbol myristate acetate (PMA) (Sigma Aldrich) plus 0.5 µg/ml ionomycin (Sigma Aldrich), and then incubated with GolgiStop (BD Biosciences) for 5 hours at 37°C in a humidified atmosphere of 5% CO_2_. After washing twice with 3% FCS-supplemented RPMI-1640 and blocking of Fc receptors, staining of cell surface markers was performed as described above. The cells were fixed and permeabilized with BD Cytofix/Cytoperm solution (BD Biosciences) according to manufacturer's instruction, the cells were stained with PE-labeled anti-IFN-γ (XMG1.2), anti-IL-4 (11B11), anti-IL-10 (JES5-16E3) or anti-IL-17 (TC11-18H10.1) mAb, and then washed twice with BD Perm/Wash buffer. Cells were analyzed on a FACSCanto II with CellQuest software (BD Biosciences).

### Quantitative PCR

Three hours after Pd challenge, ear auricles were isolated. Total RNA from ear auricles was extracted using the RNeasy lipid tissue kit (QIAGEN, Hilden, Germany) according to the manufacturer's instruction. Two µg of total RNA was reverse transcribed using SuperScript III with Oligo (dT)_12–18_ as the primer (Invitrogen, Carlsbad, CA, USA). The reverse transcribed sample was diluted 1∶10 and used as a cDNA template. Quantitative (real-time) PCR was carried out in a DNA chromo 4 (Bio-Rad Laboratories, Hercules, CA, USA) according to the manufacturer's instruction. Specific PCR primers were as follows: pan-H60 (it detects H60a, b, and c): sense 5′-gtg ctc agt gaa tgg aaa gac-3′, antisense 5′- ggc att tct cta aag tgt g-3′; pan-Rae-1: sense 5′-atg gcc aag gca gca gtg acc aag cgc cat-3′, antisense 5′-tca cat tgc aaa tgc aaa tgc aaa taa taa-3′; GAPDH: sense 5′-tga agg tcg gtg tga acg gat ttg gc-3′, antisense 5′-cat gta ggc cat gag gtc cac cac-3′. SYBR green was used for quantification of the amplified DNA. The cycling conditions were: 95°C for 3 min, followed by 40 cycles of 95°C 15 sec, 60°C for 30 sec and 72°C for 1 min. At the end of the run, the melting temperature of the amplified product was measured to confirm its homogeneity. Data were analyzed by using Opticon Monitor version 3 software (Bio-Rad laboratories).

### Statistics

Student's *t*-test was used for analysis of differences. Values of *P*<0.05 were considered to indicate statistical significance. Percentages shown in flow cytometry data were statistically analyzed in three independent experiments.

## Results

### Metal allergy is transferred to recipient nude mice by adoptive transfer of lymphocytes from disease model mice

**Figure 1:**
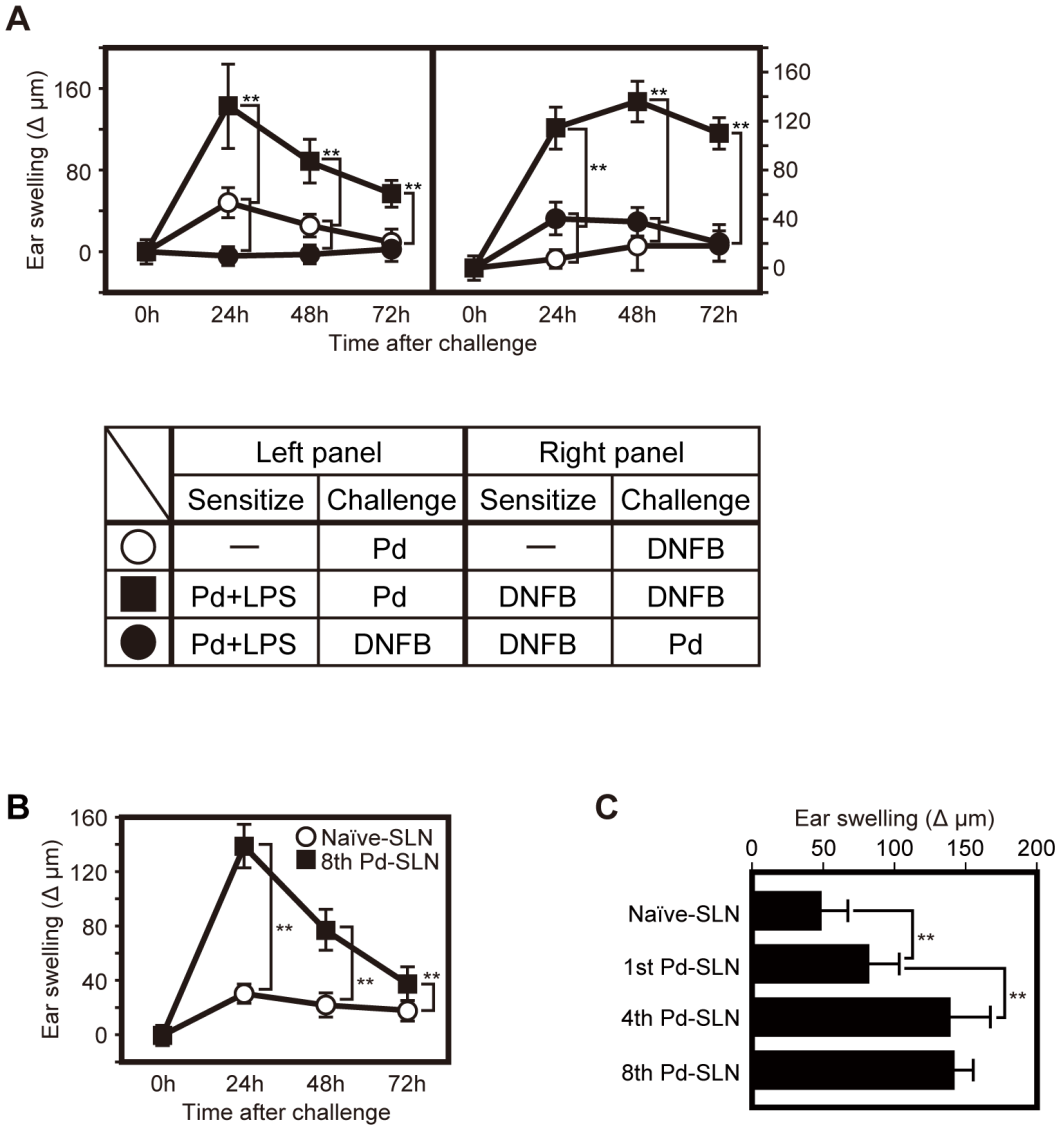
Pd allergy is induced by hapten-specific responseand transferable through pathogenic T cell transfer. (A) Ear thickness was measured before challenge and at the indicated time point after challenge. CHS reaction was elicited in Pd plus LPS-sensitized mice (left panel) or DNFB-sensitized mice (right panel), as described. As negative controls (open circles), mice were i.p. injected with PBS (left panel) or vehicle alone (AOO) was applied (right panel) followed by challenge with Pd or DNFB. Data represent means ± SD of 10 ear samples and similar results were observed in two independent experiments. Asterisks (11) indicates statistical significance (*P*<0.01) between Pd-challenged mice and DNFB-challenged mice. Lower panel shows combination of sensitization and challenge. (B) BALB/c nude mice were adoptively transferred with naïve SLN cells (open circle) or 8th Pd-SLN cells (filled square) as described. Seven days after the transfer, recipient mice (n = 5–8) were challenged with 10 nmol of Pd. The ear swelling was measured as described in(A). Data are represented as the mean ± SD. Asterisks (11) indicates statistical significance (*P*<0.01). (C) Ear swelling at 24 hours after Pd challenge was compared between 1st, 4th, and 8th adoptive transfer of Pd-SLN cells. Data are represented as the mean ± SD. Asterisks (11) indicates statistical significance (*P*<0.01).

To examine whether induction of metal allergies are hapten-specific, allergic responses were induced in Pd plus LPS-sensitized mice by challenge with Pd or DNFB solution (Fig. S1A). Specifically, 10 days after sensitization by i.p. injection of Pd solution in combination with LPS, mice were challenged by i.d. injection of Pd or application of DNFB solution into ear auricles. Sensitized, Pd-challenged mice exhibited an allergic response (ear swelling) (closed square, left panel, Fig. 1A). In contrast, sensitized, DNFB-challenged mice (closed circle, left panel, Fig. 1A) did not exhibit the ear swelling response. Although slight ear swelling of unsensitized ( = vehicle-administered), Pd-challenged mice was noted (open circle, left panel, Fig. 1A), these reactions appear to be due to non-specific inflammation as DNFB-sensitized, Pd-challenged mice exhibited a similar response (closed circle, right panel, Fig. 1A). In addition, DNFB-sensitized mice were evaluated for the induction of an allergic response to challenge with Pd or DNFB (right panel, Fig. 1A). Inflammation was not observed in unsensitized, DNFB-challenged mice (open circle, right panel, Fig. 1A). While DNFB-sensitized, DNFB-challenged mice exhibited an ear swelling response (closed square, right panel, Fig. 1A), DNFB-sensitized, Pd-challenged mice did not (closed circle, right panel, Fig. 1A). Thus, we found that Pd allergy is hapten-specific and that Pd-specific immunological responses develop in mice.

To determine whether αβ T cells contribute to the development of metal allergy in mice, we evaluated induction of Pd allergy in nude mice which lack intrathymic T cell development, and observed no evidence of induction of Pd allergy. Because γδ T cells and NK cells are present in nude mice, the lack of allergic response in nude mice indicates that thymic-derived αβ T cells are important for the development of metal allergy (data not shown). Furthermore, when wild-type (WT) mice were left unsensitized, sensitized with Pd alone, or LPS alone and then challenged with i.d. injection of the same Pd dose, we did not observe ear swelling (data not shown).

It has been reported that Ni allergy is transferrable through lymphocyte adoptive transfer [32]. Thus, we predicted that if pathogenic T cells existed and were essential for the development of Pd allergy, then disease would be transferred into naïve nude mice by adoptive lymphocyte transfer. To address this possibility, we isolated SLN cells from Pd allergy-induced BALB/c mice, and adoptively transferred these cells (Pd-SLN cells) into naïve, syngeneic nude mice. The experimental design is depicted in Fig. S1B and C. As a control, SLN cells from naïve BALB/c mice (naïve-SLN cells) were adoptively transferred into naïve nude mice (naïve-SLN transferred mice). Seven days after the transfer, recipient mice were challenged with an i.d. injection of Pd without prior sensitization. Interestingly, 11 out of 19 Pd-SLN transferred-mice (58%) showed significant ear swelling in response to Pd challenge (Table 1) compared with naïve-SLN transferred mice (*P*<0.05), indicating that Pd-SLN cells can provoke inflammation in response to Pd challenge in recipient nude mice. In addition, B cell-depleted lymphocytes isolated from Pd-SLN cells could also elicit an ear swelling reaction in response to Pd challenge (data not shown). These results suggest that T cells are the lymphocyte subset responsible for ear swelling in recipients after adoptive transfer of polyclonal lymphocytes.

**Table 1:** Increase in disease development by repeat adoptive transfer of lymphocytes from disease-bearing mice.

Transferred cell	Ratio of ear swelling-positive to negative mice at 24 hours post Pd challenge
Naïve-SLN	0% (0/10)*
None	0% (0/4)
1st Pd-SLN	58% (11/19)
4th Pd-SLN	75% (3/4)
8th Pd-SLN	95% (20/21)

* SLN cells from naïve BALB/c mice were adoptively transferred into naïve nude mice.

To enrich for endogenous pathogenic T lymphocytes, we repeated the adoptive transfer and Pd challenge procedures until more than 90% of recipient mice exhibited ear swelling. We selectively isolated Pd-SLN cells from recipient nude mice in which ear swelling was elicited, and then transferred these cells into a new batch of naïve nude mice. αβ T cells were more than 99.9% in Pd-SLN T cells from recipient nude mice. After the 4th adoptive transfer, ear swelling was elicited in 3 out of 4 mice following Pd challenge (75%) (Table 1). After the 8th transfer, 20 out of 21 mice (95%) exhibited significant ear swelling (Table 1), which reached a peak at 24 hours post-Pd challenge (Fig. 1B, closed squares). Ear thickness declined to the basal level at 96 hours after Pd challenge (data not shown). In contrast, ear swelling was only marginal in nude mice adoptively transferred with naïve-SLN cells (Fig. 1B, open circles). Although the period of ear swelling was not prolonged, the intensity was increased by sequential rounds of adoptive transfer (Fig. 1C).

**Figure 2:**
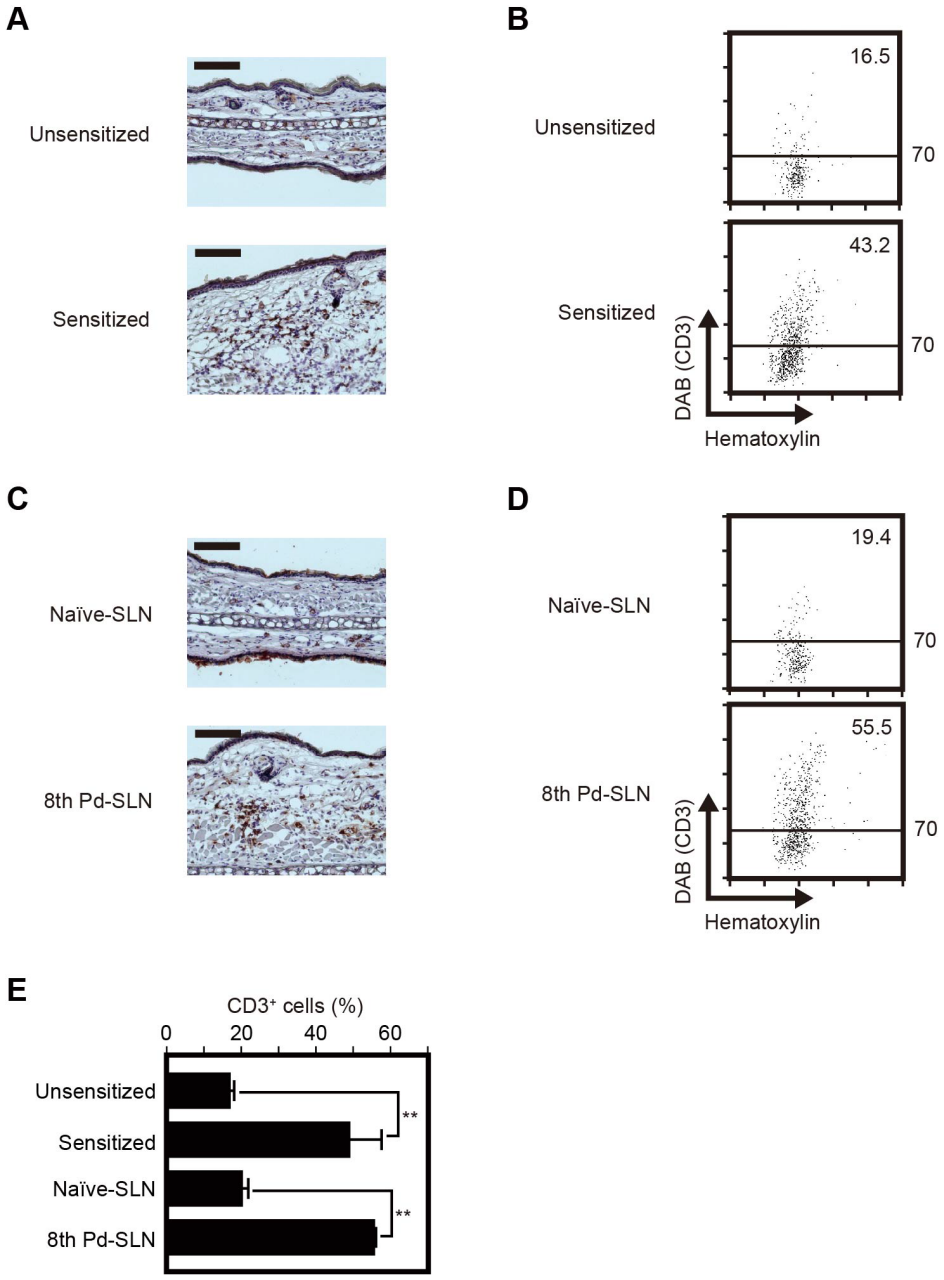
Pathogenic T cells are present in a mouse model of metal allergy. Immunohistochemical analysis of the ear auricles at 24/c mice (A) and 8th Pd-SLN cells-transferred nude mice (C). Ear sections were stained by anti-CD3ε mAb and visualized by DAB. The sections were counterstained with hematoxylin. The scale bars indicate 100 µm. (B) Quantification of CD3ε-positive T cells shown in panel A for WT BALB/c mice and (D) in panel C for 8th Pd-SLN cells-transferred nude mice. Representative images from each group were analyzed and presented as scattergrams using HistoQuest software. X- and Y-axis are intensity of hematoxylin and DAB, respectively. Numbers shown in the scattergrams indicates percentage of CD3ε-positive T cells. Similar results were obtained in three independent experiments. (E) CD3ε-positive T cells infiltrated in ear auricles were statistically analyzed using results from image cytometry. Data represent means ± SD of 6 ear samples. Asterisks (11) indicate statistical significance (*P*<0.01). Similar results were obtained in three independent experiments.

Immunohistochemical staining was conducted at 24 hours after the Pd challenge in order to identify infiltrating immune cells. CD3ε^+^ T cells were found in the inflammatory lesions in Pd plus LPS-sensitized WT mice significantly more than unsensitized WT mice (Fig. 2A). Quantitative analysis by image cytometry indicated that CD3ε^+^ T cells in the inflammatory site were increased from 16.5% to 43.2% by sensitization with Pd plus LPS in WT mice (Fig. 2B). As shown in Fig. 2C and D, immunological staining at 24 hours post-challenge showed that CD3ε^+^ T cells were also infiltrating into the inflammatory lesions in nude mice with 8th transfer Pd-SLN cells (55.5% of cells) compared with that in nude mice with naïve-SLN cells (19.4% of cells). Percentages of CD3ε^+^ T cells were analyzed from multiple experiments and statistical analyses were performed (Fig. 2E). In unsensitized, Pd-challenged WT mice 17.2±1.0% CD3ε^+^ T cells were found, 49.2±8.5% in Pd plus LPS-sensitized, Pd-challenged WT mice, 20.5±1.5% for naïve-SLN cells-transferred nude mice, and 55.8±0.4% for 8th Pd-SLN cells-transferred nude mice. These results indicate that T cells are responsible for the induction of metal allergy, and that eight rounds of sequential adoptive transfer are required to selectively enrich for the pathogenic cell type.

### CD8^+^ T lymphocytes contribute to the development of metal allergy

**Figure 3:**
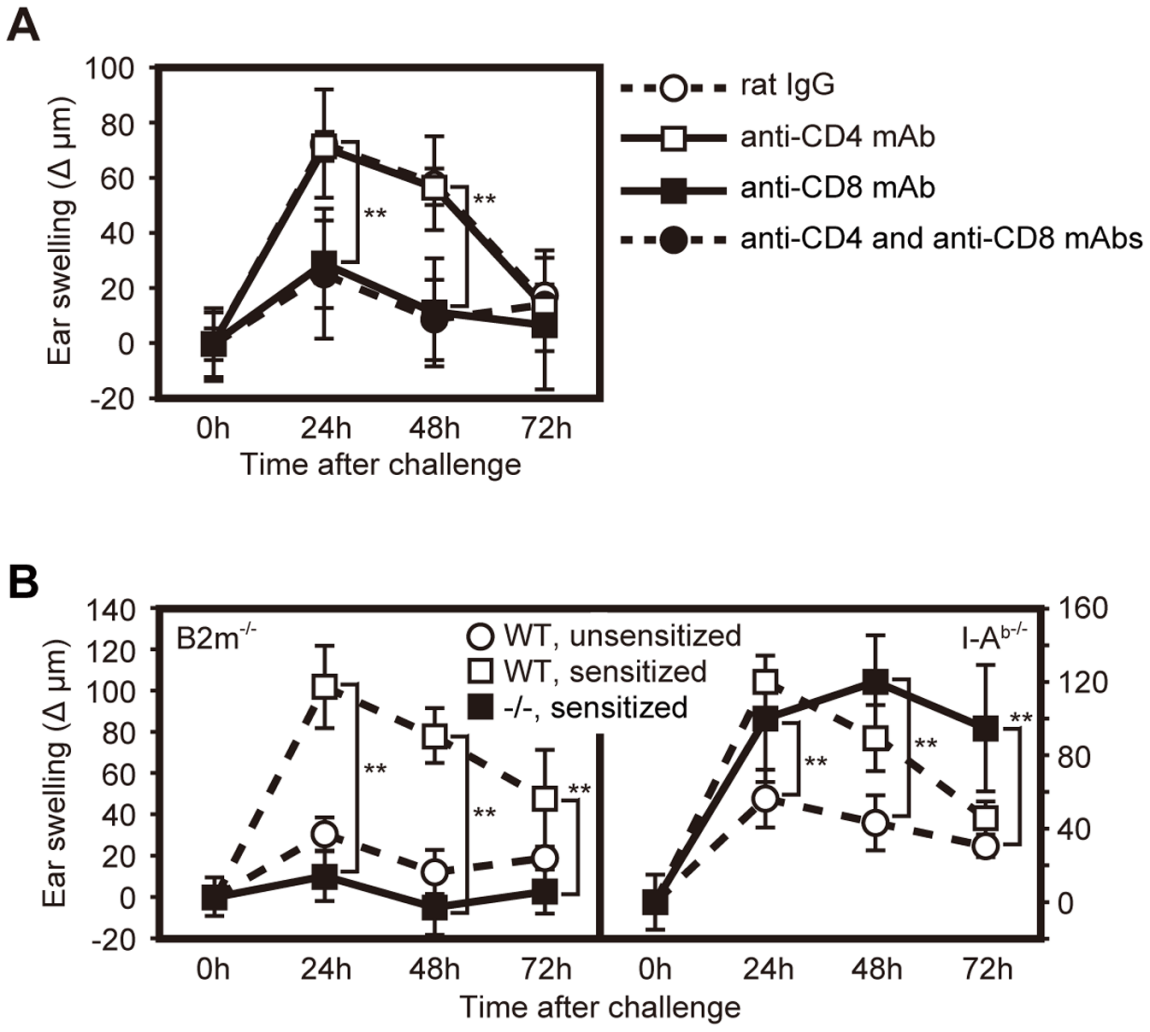
CD8^+^ T cells are required for the development of Pd allergy. (A) Ear swelling induced by Pd allergy was evaluated in anti-CD4 and/or anti-CD8 mAb-administered mice. In these mice, CD4^+^ and/or CD8^+^ T cells were depleted, respectively. As a control, rat IgG was administered instead of anti-CD4 or anti-CD8 mAb. Ear thickness was measured as described in Fig. 1A. Data are presented as the mean ± SD of 10 ear samples, and similar results were observed in three independent experiments. Asterisks (11) indicate statistical significance (11*P*<0.01) between anti-CD8 mAb-administered mice and control mice. (B) Pd allergy was elicited in B2m–deficient mice (left panel) or I-A^b^-deficient mice (right panel) as described. As experimental controls, C57BL/6 WT mice (n = 5) were sensitized by i.p. injection with 2.5 µmol Pd and 2.5 µg LPS (sensitized) or PBS (unsensitized). Ear thickness was measured as described in Fig. 1A. Data represent the mean ± SD of 8–10 ear samples, and similar results were observed in three (B2m-deficient) or two (I-A^b^-deficient) independent experiments. Asterisks (11) indicates statistical significance (*P*<0.01) between sensitized B2m-deficient (−/−) mice and sensitized WT mice or between sensitized I-A^b^-deficient (−/−) mice and unsensitized WT mice.

Because we found that pathogenic T cells infiltrated into inflammatory lesions of nude mice that had received transferred T cells derived from mice with metal allergic disease, we further investigated whether CD4^+^ or CD8^+^ T cells were preferentially involved in Pd allergy. To this end, we depleted CD4^+^ or CD8^+^ T cells from 8th Pd-SLN transferred nude mice by administration of anti-CD4 or anti-CD8 mAbs in vivo. Although ear swelling was observed in mice depleted of CD4^+^ T cells as well as mice administered rat IgG, ear swelling was significantly inhibited in mice depleted of CD8^+^ T cells alone or of both CD8^+^ and CD4^+^ T cells (Fig. 3A).

To confirm the role of CD8^+^ T cells in metal allergy, we induced Pd allergy in B2m-deficient mice and MHC class II I-A^b^-deficient mice that lack MHC class I and MHC class II, respectively. Of note, CD8^+^ T cells are impaired in B2m-deficient mice and CD4^+^ T cells are impaired in I-A^b^-deficient mice. We did not observe significant ear swelling in B2m-deficient mice, whereas I-A^b^-deficient mice did exhibit an ear swelling in response to Pd (Fig. 3B). The maximum ear thickness in I-A^b^-deficient mice was comparable with that in WT mice; however, the response was prolonged in I-A^b^-deficient mice (Fig. 3B). These results indicate that, similar to other strong experimental haptens, MHC class I-restricted CD8^+^ T cells are required for the onset of Pd allergy [31].

### IFN-γ production in CD8^+^ T cells contributes to the development of Pd allergy

**Figure 4:**
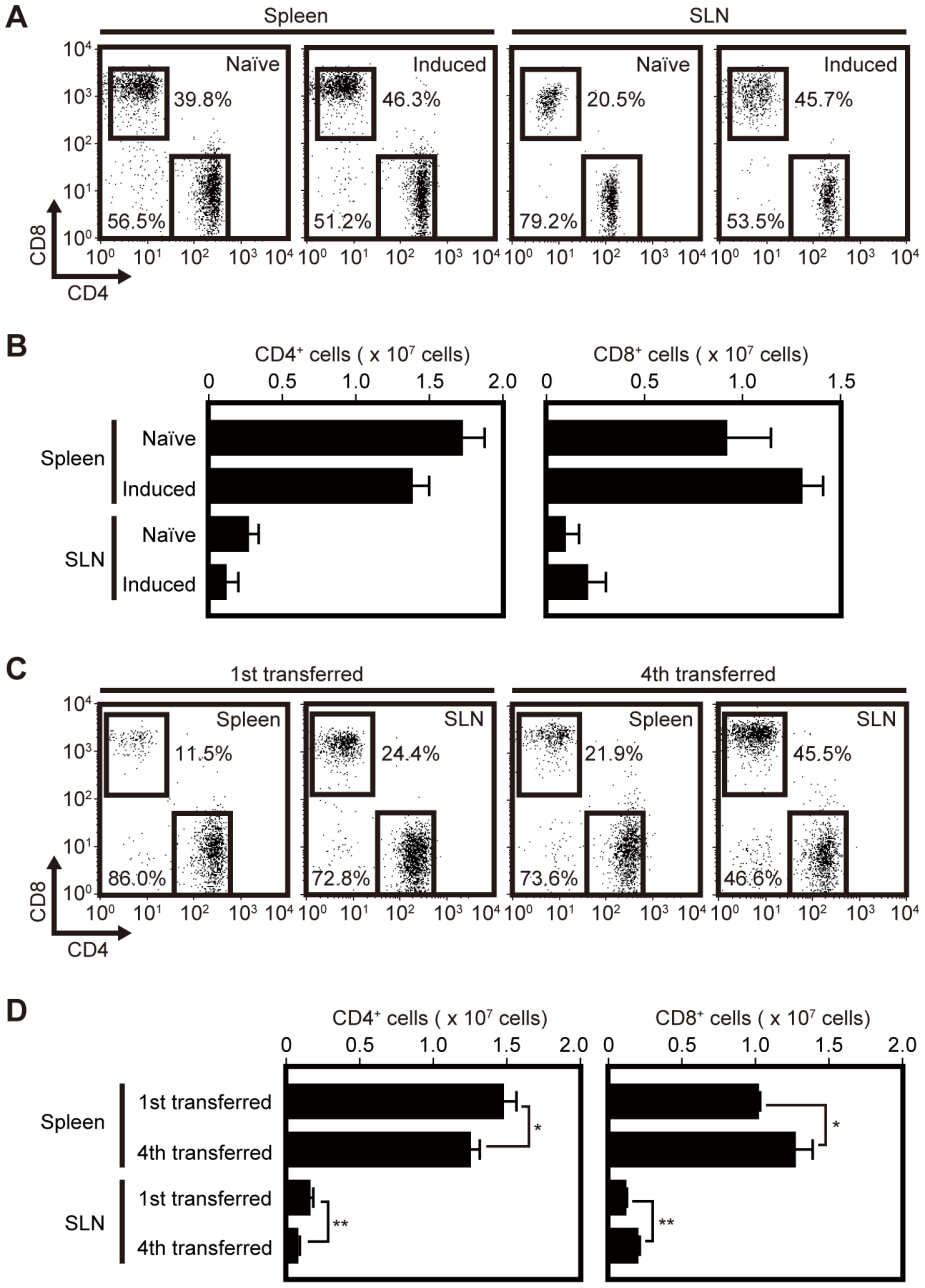
CD8^+^ T cells are enriched in draining lymph nodes following the development of metal allergy. (A) Before (Naïve) and after (Induced) induction of metal allergy, the percentage of CD4^+^ or CD8^+^ T cells present in splenocytes or SLN cells from WT mice was analyzed by flow cytometry. Data shown were obtained from TCR β^+^-gated, viable cells. Percentages shown in (A) are one of representative results. Similar results were obtained in three independent experiments. (B) Cell numbers of CD4^+^ or CD8^+^ T cells in (A) were calculated as follows: absolute cell numbers x percentages of each subset (by flow cytmetry analysis). (C) The percentage of CD4^+^ or CD8^+^ T cells present in splenocytes or SLN cells from 1st and 4th adoptive transferred recipient mice was analyzed by flow cytometry. Of note, αβ^+^ T cells were not detected in the SLN or spleen from naïve nude mice. Data shown were obtained from TCR β^+^-gated, viable cells. Percentages shown in (C) are one of representative results. Similar results were obtained in three independent experiments. (D) Cell numbers of CD4^+^ or CD8^+^ T cells in (C) were calculated as follows: absolute cell numbers x percentages of each subset (by flow cytmetry analysis). Asterisks (1 or 11) indicate statistical significance (0.01<1*P*<0.05 or 11*P*<0.01, respectively) between 1st adoptive transferred sample and 4th sequential adoptive transferred samples.

To examine whether CD8^+^ T cells accumulate in draining lymph nodes, we analyzed accumulation of αβ T cell populations in SLN cells or splenocytes from WT mice before and after induction of Pd allergy (Fig. 4A and B). Whereas T cell numbers slightly increased in splenocytes following induction of Pd allergy, percentages of CD8^+^ T cells in SLN increased more than twofold (Fig. 4A). Furthermore, we analyzed the T cell population in splenocytes and SLN cells from the recipient nude mice after the 4th adoptive transfer (Fig. 4C and D). Approximately 24% of CD8^+^ T cells and 73% of CD4^+^ T cells were present in SLN of 1st transferred mice (Fig. 4C and D). In contrast, 46% of CD8^+^ T cells and 47% of CD4^+^ T cells were present in SLN from the recipient mice after the 4th adoptive transfer (Fig. 4C and D). These results indicate that CD8^+^ T cells, rather than CD4^+^ T cells, preferentially accumulated in the draining lymph nodes in response to Pd challenge.

**Figure 5:**
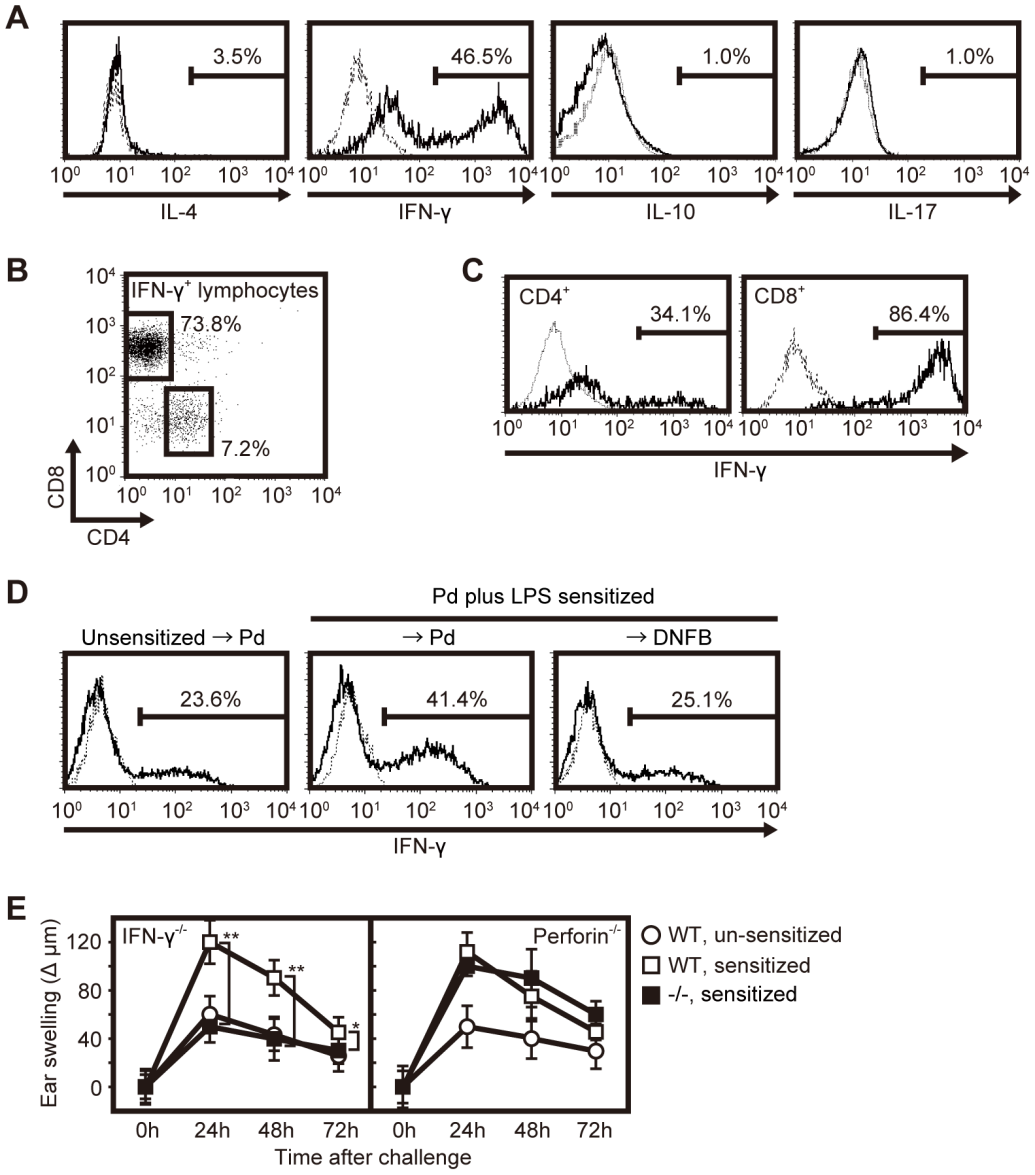
IFN-γ from CD8^+^ T cells contributes to the development of metal allergy. (A) Fifteen hours after Pd challenge, 8th Pd-SLN cells were isolated and analyzed for cytokine production (IL-4, IFN-γ, IL-10, and IL-17). Data presented were obtained from total lymphocytes. (B) IFN-γ^+^ T cell subset analysis. Cells were prepared and stained with anti-CD4, -CD8 and -IFN-γ mAbs, then analyzed by flow cytometry. Percentages of T cell subset in IFN-γ positive lymphocytes were as follows; 7.7±0.1% for CD4^+^ T cells and 72.4±1.4% for CD8^+^ T cells. (C) IFN-γ production from each subset of T cells in the 8th Pd-SLN. The cell population analyzed is indicated in each panel. (D) Comparison of IFN-γ production from CD8^+^ T cells in Pd- or DNFB-challenge of Pd plus LPS-sensitized mice. As a control, unsensitized mice were challenged with PdCl_2_. The cell population analyzed is indicated in each panel. Percentages of IFN-γ positive cells in CD8^+^ T cells were as follows; 23.2±0.6% for unsensitized, Pd-challenged mice, 42.3±1.3% for Pd plus LPS-sensitized, Pd-challenged mice, and 23.7±2.0% for Pd plus LPS-sensitized, DNFB-challenged mice. (A, C and D) The dotted line indicates isotype-matched control Ig staining. (A–D) Similar results were obtained in three independent experiments. (E) Pd allergy was induced in IFN-γ-deficient mice (left panel) or perforin-deficient mice (right panel) as described. Experimental controls were performed as described in Fig. 3B. Ear swelling was measured as described in Fig. 1A. Data represent means ± SD of 10 ear samples. Asterisks (1 or 11) indicate statistical significance (0.01<1*P*<0.05 or 11*P*<0.01, respectively) between sensitized deficient (−/−) mice and sensitized WT mice. Similar results were observed in three (IFN-γ-deficient) or two (perforin-deficient) independent experiments.

Cytokines have also been reported to play important roles in metal allergy [8], [11], [33]. Thus, we examined production of IL-4, IFN-γ, IL-10 or IL-17 from lymphocytes of 4th Pd-SLN transferred nude mice. Pd-SLN cells were isolated 15 hours after Pd challenge and restimulated with PMA plus ionomycin in vitro. We found that 46.5% of Pd-SLN cells produced IFN-γ, while in contrast, only 3.5% of Pd-SLN cells produced IL-4 (Fig. 5A and Fig. S3A). Furthermore, neither IL-10 nor IL-17 was produced by these cells in response to this stimulation (Fig. 5A and Fig. S3A). To determine which T cell subset contributes to IFN-γ production, we analyzed the ratio of CD4^+^ T versus CD8^+^ T cells in IFN-γ positive lymphocytes. Approximately 73.8% of IFN-γ^+^ cells were CD8^+^ T cells (Fig. 5B), furthermore, 86.4% of CD8^+^ T cells produced IFN-γ (Fig. 5C and Fig. S3B).

Moreover, we evaluated whether the production of IFN-γ from CD8^+^ T cells is a sensitized hapten-specific reaction. Fifteen hours after administration of Pd or DNFB into ear auricles of Pd plus LPS-sensitized WT mice, lymphocytes were isolated from SLN cells. As shown in Fig. 5D, 25.1% of CD8^+^ T cells were IFN-γ-positive in Pd plus LPS-sensitized, DNFB-challenged mice, and 23.6% of CD8^+^ T cells were also IFN-γ-positive in unsensitized, Pd-challenged mice. In contrast, 41.4% of CD8^+^ T cells were IFN-γ-positive in Pd plus LPS-sensitized, Pd-challenged mice. Of note, 8.2% of CD8^+^ T cells in naïve SLN were IFN-γ^+^ cells, and 23.6% of CD8^+^ T cells in SLN of unsensitized Pd-challenged mice (Table S1). This would indicate that CD8^+^ T cells are primed to produce IFN-γ within 15 hours after Pd challenge in unsensitized mice. However, 41.4% of CD8^+^ T cells in SLN from Pd plus LPS-sensitized, Pd-challenged mice were IFN-γ^+^ cells, and these percentages are significantly higher than unsensitized mice (Table S1). These results indicate that ear swelling response is due to a hapten-specific secondary immunological response and that IFN-γ^+^ CD8^+^ T cells are increased during a Pd-specific response.

Because IFN-γ is the main cytokine produced by CD8^+^ T cells responding to Pd allergy, we evaluated ear swelling in mice deficient for IFN-γ and found no ear swelling when Pd allergy was induced (Fig. 5E). CD8^+^ T cells are known to be cytotoxic T cells (CTLs) that kill target cells by release of perforin and granzymes [34]–[36]. Given that CD8^+^ T cells were implicated in the development of Pd allergy, we also examined ear swelling in perforin-deficient mice; however, perforin-deficient mice did exhibit ear swelling in response to Pd challenge (Fig. 5E). These results indicate that IFN-γ production, but not release of perforin from CD8^+^ T cells, is required for the induction of Pd allergy.

### NKG2D expressed on CD8^+^ T cells plays an important role in Pd allergy

**Figure 6:**
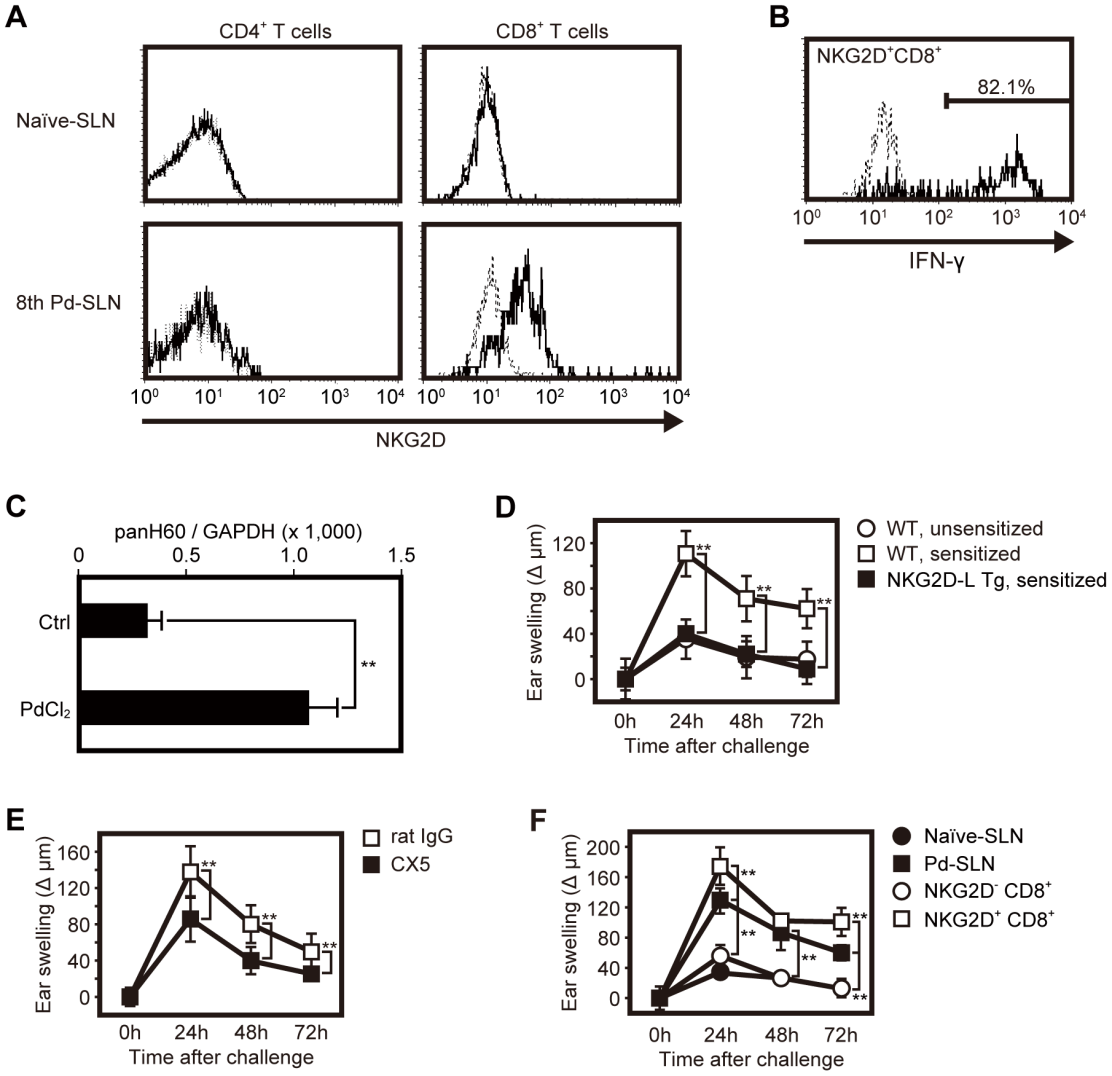
Expression of NKG2D on T cells contribute to the development of metal allergy. (A) Ninety-six hours after Pd challenge, 8th Pd-SLN cells were prepared and analyzed for expression of CD4, CD8, and NKG2D by flow cytometry. As a control, naïve-SLN cells were analyzed. The dotted line indicates staining of isotype-matched control Ig. Similar results were obtained in eight independent experiments. (B) Fifteen hours after elicitation, 8th Pd-SLN cells were obtained and IFN-γ production by NKG2D^+^ CD8^+^ cells was analyzed. The dotted line indicates staining with isotype-matched control Ig. Data shown is one of representative results. Similar results were obtained in three independent experiments. Percentage of IFN-γ positive cells in NKG2D^+^ CD8^+^ T cells was 83.6±2.1%. (C) Three hours after i.d. administration of PdCl_2_ (or PBS as a control) into ear auricles, total RNA was extracted from the ear auricles and cDNA was synthesized. Expression of the NKG2D ligand, pan-H60, transcripts were quantified by real-time PCR and normalized with GAPDH. Data represent means ± SD of 5 ear samples. Asterisks (11) indicate statistical significance (*P*<0.01) between PdCl_2_-injected and control samples. Similar results were obtained in three independent experiments. Ear swelling induced by Pd allergy was evaluated in NKG2D ligand transgenic (Tg) mice (D), anti-NKG2D mAb-administered mice (E), and NKG2D positive- or negative-CD8^+^ T cell transferred nude mice (F). Experimental controls in (D) were performed as described in Fig. 3B. In (E), rat IgG was administered instead of anti-NKG2D mAb as a control. In (F), naïve-SLN or Pd-SLN cells were transferred into naïve nude mice as negative or positive control, respectively. In (F), 3×10^5^ cells of each sample were adoptively transferred into naïve nude mice. Ear thickness was measured as described in Fig. 1A. Data are represented as the mean ± SD of 8–10 ear samples, and similar results were observed in two (D and F) or three (E) independent experiments. Asterisks (11) indicates statistical significance (11*P*<0.01).

Costimulatory molecules are required for cytokine production from T cells. Given that CD8^+^ T cells are crucial for the development of Pd allergy (Fig. 3 and Fig. 4), we hypothesized that NKG2D on CD8^+^ T cells may be important for the pathogenesis of Pd allergy. Indeed, CD8^+^ T cells of 8th transfer Pd-SLN mice express NKG2D on their surface while naïve-SLN cells do not (Fig. 6A), indicating that the NKG2D molecule is upregulated on CD8^+^ T cells during enrichment by sequential adoptive transfer. We also measured whether NKG2D^+^ CD8^+^ T cells produce IFN-γ in response to induction of Pd allergy. Approximately 82% of the NKG2D^+^ CD8^+^ T cells produced IFN-γ (Fig. 6B). In addition, H60, one of the NKG2D ligands, was upregulated at the site of Pd inflammation (Fig. 6C). The above results prompted us to investigate whether NKG2D on CD8^+^ T cells is directly involved in development of Pd allergy. To this end, we induced Pd allergy in *Raet1e* transgenic mice. RAE-1 is a known NKG2D ligand and excess expression of RAE-1 strongly downmodulates expression of NKG2D [26]. Thus, NKG2D expression is decreased in *Raet1e* transgenic mice (Fig. S2). In these mice, induction of Pd allergy as measured by ear swelling was negligible (Fig. 6D).

### Neutralizing NKG2D ameliorates Pd-induced ear swelling

To investigate whether a neutralizing anti-NKG2D mAb (hybridoma clone: CX5) might be useful as a therapeutic reagent for Pd allergy, we performed an anti-NKG2D mAb administration study. Blocking of NKG2D significantly ameliorated Pd-induced ear swelling in 8th transfer Pd-SLN nude mice (Fig. 6E). In addition, we wanted to confirm whether NKG2D^+^ CD8^+^ T cells are necessary for induction of Pd allergy. NKG2D^+^ CD8^+^ T cells or NKG2D^−^ CD8^+^ T cells were purified from Pd plus LPS-sensitized mice and adoptively transferred into naïve nude mice. Transfer of NKG2D^+^ CD8^+^ T cells significantly induced ear swelling following Pd challenge compared to transfer of Pd-SLN cells (open squares, Fig. 6F). In contrast, ear swelling was not observed in NKG2D^−^ CD8^+^ T cells-transferred mice (open circles, Fig. 6F). These results indicate that NKG2D^+^ CD8^+^ T cells function as pathogenic T cells that produce IFN-γ in response to Pd allergy in vivo, and thus, NKG2D is a promising target for the treatment of metal allergy.

## Discussion

In this study we found that a metal allergic disease, Pd allergy, was transferred into recipient nude mice by adoptive transfer of lymphocytes from disease-bearing mice. Although the period of inflammation was not extended, the frequency and severity of symptoms were increased by successive rounds of adoptive transfer (Fig. 1). Moreover, CD8^+^ T cells are required for the development of Pd allergy because Pd allergy is not developed in mice depleted of CD8^+^ T cells or in B2m deficient mice (Fig. 3). CD8^+^ T cells preferentially accumulated in the draining lymph nodes of Pd-SLN sequentially transferred recipient mice, and these cells predominantly expressed NKG2D (Fig. 4 and Fig. 6). Furthermore, the NKG2D^+^ CD8^+^ T cells produced IFN-γ, an effector molecule that plays a key role in the development of Pd allergy (Fig. 5 and Fig. 6).

Recently, it was reported that Ni directly stimulates human TLR4 to produce inflammatory cytokines, which may be crucial for induction of contact allergy to the metal [37]. We also detected TNF-α in the culture supernatant of human PBMCs stimulated by Ni; however, not from Pd-stimulated cells (unpublished observation). Pd allergy was only induced by sensitization with LPS, suggesting that the mechanism for the development of metal allergy might vary with the type of metal, and that signals from TLR4 by LPS stimuli might be required for induction of metal allergy.

Limited TCRs were reportedly observered in human T cells from patients with metal allergy [38], [39], because metal ions functions as non-classical haptens. We also found that the oligoclonal Vα TCR repertoire subsets were found to be increased in our animal model (unpublished observation). Thus, our animal model has advantages in the elucidation of pathogenic T cell function and is therefore a valuable tool for exploration of the molecular mechanisms underlying the development of metal allergy.

We found that pathogenic CD8^+^ T cells secrete IFN-γ as an effector molecule for induction of Pd allergy. Consistent with our results, IFN-γ from CD8^+^ T cells is one of effector molecules in the DTH response to DNFB [40], [41]. Although there are several reports of IL-4 involvement in metal allergy [9], [32], we could not detect IL-4 production from pathogenic lymphocytes in our animal model (Fig. 5A). We found that IFN-γ was mainly produced by CD8^+^ T cells which were harvested from draining lymph node 15 hours after Pd injection. Thus, we concluded that lymphocytes function as pathogenic T cells in the early phase of Pd allergy. While it is possible that lymphocytes harvested later may show different cytokine profiles, we found that IFN-γ-deficient mice exhibited a complete inhibition of ear swelling under conditions that induce Pd allergy (Fig. 5E). This finding strongly suggests that IFN-γ secretion from pathogenic CD8^+^ T cells plays an important role in development of Pd allergy.

As shown in Fig. 5E, ear swelling was observed in perforin-deficient mice and thus, cytotoxicity via the perforin pathway was not implicated in induction of Pd allergy. However, a previous study reported that the CHS reaction to DNFB is mediated by hapten-specific cytotoxic lymphocytes that may use either the Fas/FasL or the perforin pathway [42], [43]. Further studies will be required to determine if the Fas/FasL pathway plays a role in the development of Pd allergy.

In this study, the sequential adoptive transfer of cells resulted in the enrichment of CD8^+^ T cells bearing NKG2D. Interestingly, NKG2D ligands were detected in the ear following Pd administration (Fig. 6C), and we found that ear swelling was not detected in NKG2D downmodulated mice following induction of Pd allergy (Fig. 6D). In addition, neutralizing NKG2D with anti-NKG2D mAb administration in vivo significantly inhibited ear swelling under conditions that induce Pd allergy (Fig. 6E). These results indicate that NKG2D^+^ CD8^+^ T cells are responsible for Pd allergy.

Previously, we demonstrated the prevention of autoimmune diabetes [26] and the immune response to hepatitis B virus [44] by blockade of NKG2D with an anti-NKG2D mAb. Our present data suggest NKG2D may be a therapeutic target in metal allergy. Furthermore, IFN-γ was produced by not only CD8^+^ T cells, but also 34.1% of CD4^+^ T cells in this model. Lack of expression of NKG2D on CD4^+^ T cells may explain the incomplete suppression of ear swelling we observed in our blocking experiment since blocking of NKG2D would not inhibit IFN-γ production from CD4^+^ T cells. Therefore it is possible that CD4^+^ T cells may contribute to the development of Pd allergy.

Administration of Pd solution into ear auricles upregulated the expression of NKG2D ligands (Fig. 6C). We detected H60 transcripts (H60a-c) by quantitative PCR. H60c is expressed specifically in skin [45] and is involved in repair of damaged skin [46]. Although *Raet1* expression was only slightly upregulated by Pd injection (data not shown), H60 molecules were found to be markedly increased. These data suggest that H60, rather than RAE-1, functions as a ligand of NKG2D in Pd allergy.

DTH reactions to Pd, which are induced by MHC class I-restricted CD8^+^ T cells, were prolonged in I-A^b^ deficient mice (Fig. 3). Consistent with our results, DTH development and immune responses were completely inhibited in the MHC class I-deficient mice and prolonged in the MHC class II-deficient mice [31], [47]–[50]. Thus, it is likely that MHC class II-restricted regulatory T cells control the development of Pd allergy mediated by MHC class I-restricted CD8^+^ T cells.

The balance between regulatory cells and effector cells is important in the onset of autoimmune disease [51]. Pathogenic cells and regulatory cells are likely present in draining lymph nodes as mouse Pd allergy develops. We selectively isolated lymphocytes from draining lymph nodes of mice with swollen ears for sequential transfer, which favored transfer of pathogenic lymphocytes. Therefore, the onset ratio of Pd allergy and the severity of ear swelling were selectively elevated by the transfer protocol.

In conclusion, we demonstrate that enrichment of mouse pathogenic T cells by sequential adoptive transfer affects the frequency and severity of development of metal allergic symptoms. IFN-γ is produced by NKG2D^+^ CD8^+^ T cells, which are essential for the development of Pd allergy, suggesting that NKG2D is a viable therapeutic target for the treatment of metal allergy.

## Supporting Information

Figure S1 **Schematic view of metal allergy induction.** (A) Metal allergy was elicited in WT mice by sensitization with i.p. of Pd plus LPS, application of DNFB, or each vehicle and challenge with i.d. of Pd or application of DNFB. (B) Metal allergy was elicited in WT mice by sensitization with Pd plus LPS and subsequent challenge with Pd. (C) SLN cells were isolated from ear swollen mice (Pd-SLNs) or naïve mice (naïve-SLNs) and adoptively transferred to naïve nude mice. The transferred nude mice were challenged and ear swollen mice were selected, and then 2nd Pd-SLNs were isolated and further transferred to naïve nude mice.
(TIF)

Figure S2 **NKG2D was downmodulated in Rae-1 transgenic mice.** Splenocytes were prepared from C57BL/6 mice (WT) or Raet1e transgenic mice (Tg) and expression of RAE-1 and NKG2D were assessed by flow cytometry.
(TIF)

Figure S3 **IFN-γ^+^ CD8^+^ cells were induced by Pd allergy.** (A) Cell numbers of each cytokine-positive lymphocytes in Fig. 5A were estimated from absolute cell numbers of SLN and flow cytmety analysis, and analyzed statistically. (B) Cell numbers of CD4^+^ or CD8^+^ T cells in Fig. 5C were estimated from absolute cell numbers of each tissue and FACS analysis, and analyzed statistically. Asterisks (11) indicates statistical significance (11*P*<0.01).
(TIF)

Table S1 **Comparison of IFN-γ^+^ cells population between Pd-sensitization state.** Fifteen hours after Pd challenge, SLN cells were isolated and analyzed for IFN-γ production. Each percentages were analyzed by flow cytometry.
(DOCX)
