# Supplementary figures and images for: NKG2D+ IFN-γ+ CD8+ T Cells Are Responsible for Palladium Allergy
Source: PLoS One. 2014 Feb 12;9(2):e86810. doi: 10.1371/journal.pone.0086810 (PMC3922723; doi:10.1371/journal.pone.0086810)

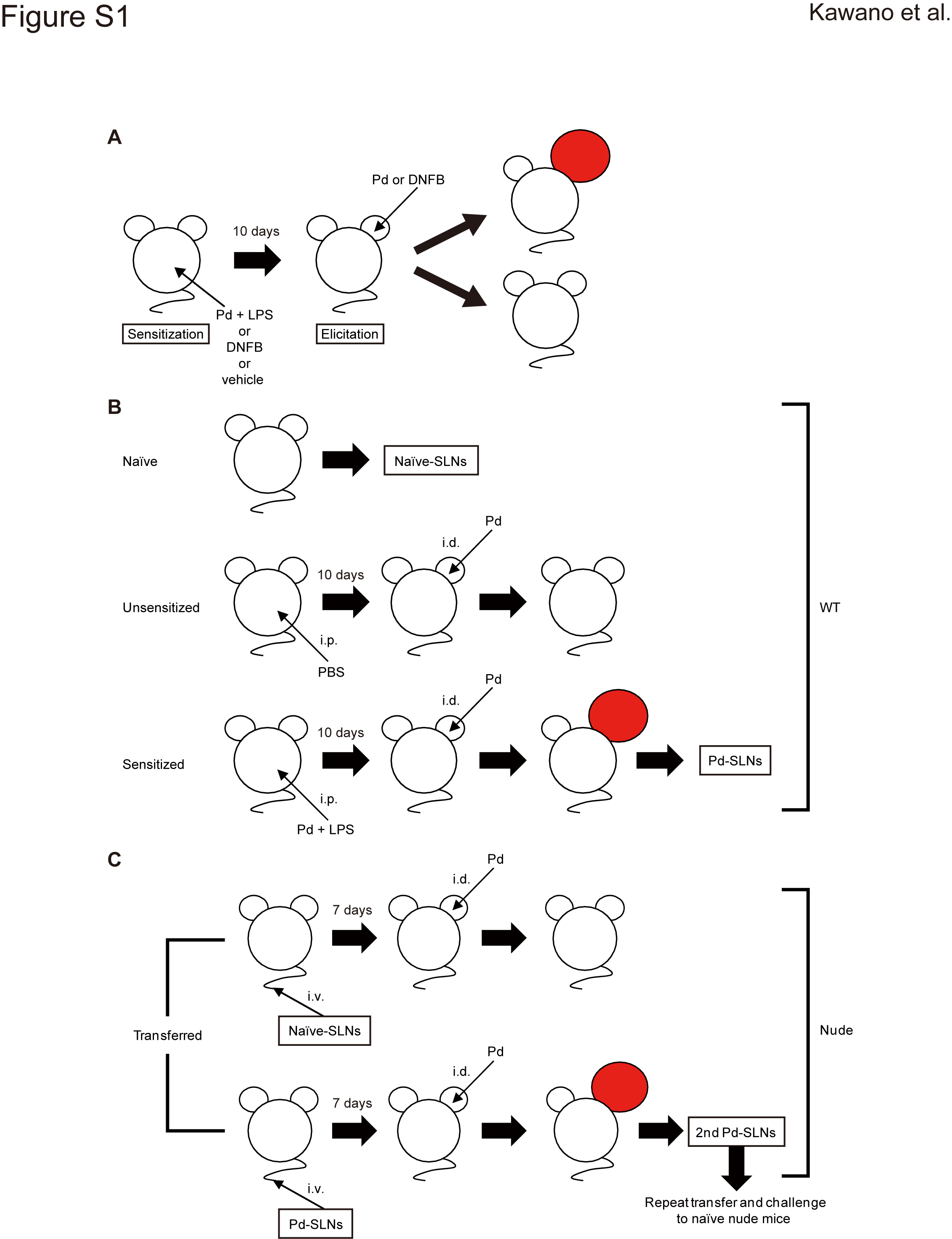

Supplement: Figure S1 — Schematic view of metal allergy induction. (A) Metal allergy was elicited in WT mice by sensitization with i.p. of Pd plus LPS, application of DNFB, or each vehicle and challenge with i.d. of Pd or application of DNFB. (B) Metal allergy was elicited in WT mice by sensitization with Pd plus LPS and subsequent challenge with Pd. (C) SLN cells were isolated from ear swollen mice (Pd-SLNs) or naïve mice (naïve-SLNs) and adoptively transferred to naïve nude mice. The transferred nude mice were challenged and ear swollen mice were selected, and then 2nd Pd-SLNs were isolated and further transferred to naïve nude mice. (TIF) [file pone.0086810.s001.tif]

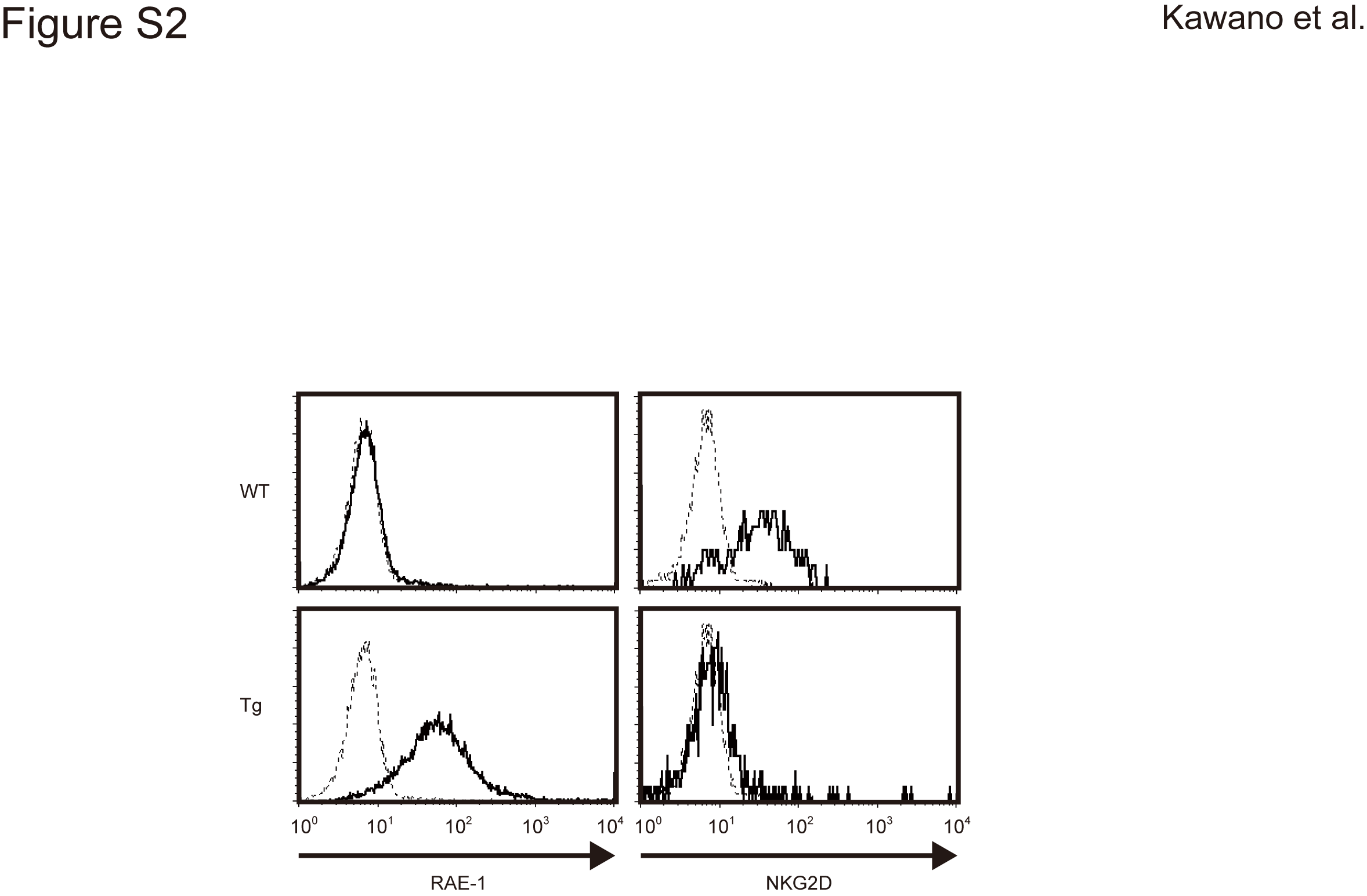

Supplement: Figure S2 — NKG2D was downmodulated in Rae-1 transgenic mice. Splenocytes were prepared from C57BL/6 mice (WT) or Raet1e transgenic mice (Tg) and expression of RAE-1 and NKG2D were assessed by flow cytometry. (TIF) [file pone.0086810.s002.tif]

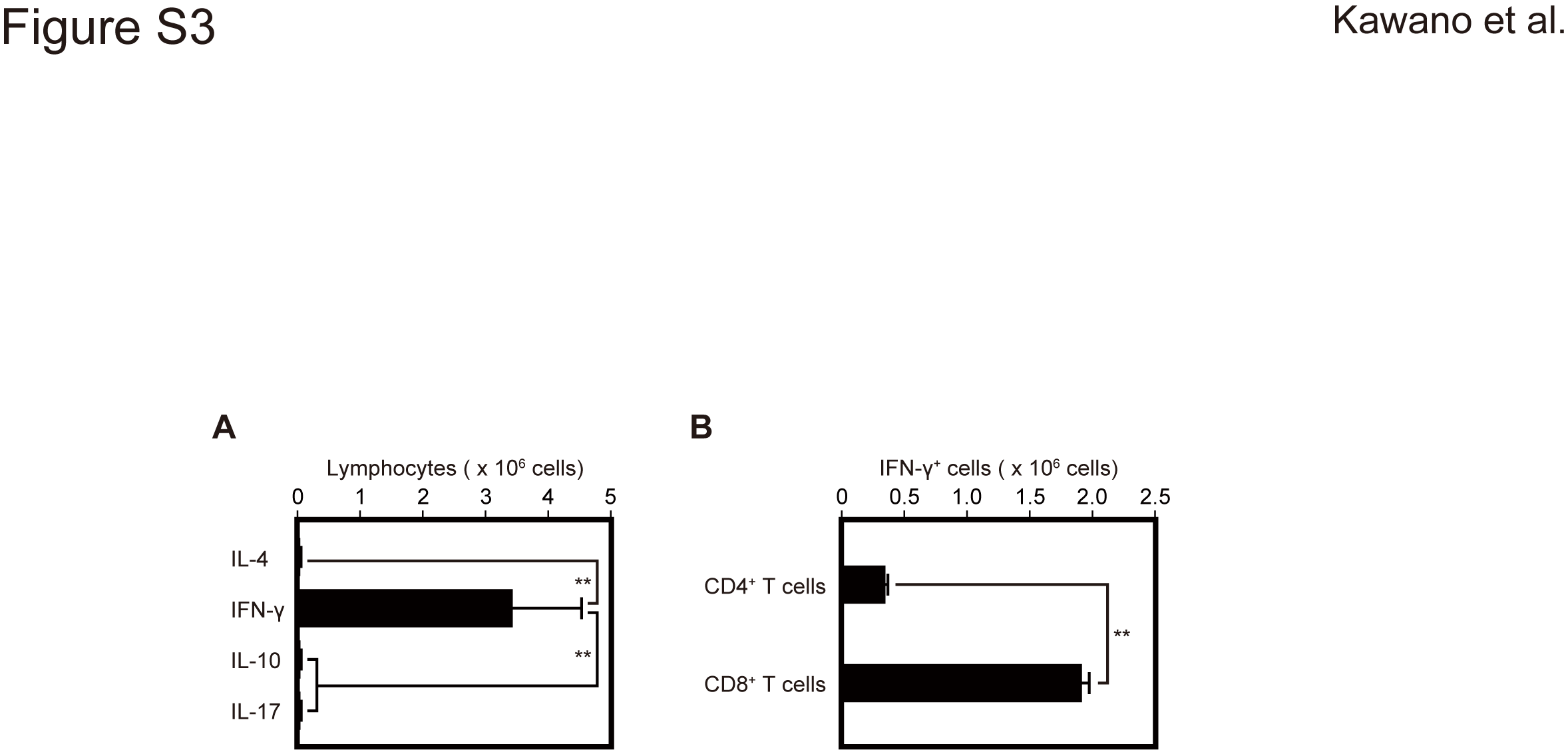

Supplement: Figure S3 — IFN-γ+ CD8+ cells were induced by Pd allergy. (A) Cell numbers of each cytokine-positive lymphocytes in Fig. 5A were estimated from absolute cell numbers of SLN and flow cytmety analysis, and analyzed statistically. (B) Cell numbers of CD4+ or CD8+ T cells in Fig. 5C were estimated from absolute cell numbers of each tissue and FACS analysis, and analyzed statistically. Asterisks (11) indicates statistical significance (11P<0.01). (TIF) [file pone.0086810.s003.tif]
