# Supplementary material for: NKG2D+ IFN-γ+ CD8+ T Cells Are Responsible for Palladium Allergy
Source: PLoS One. 2014 Feb 12;9(2):e86810. doi: 10.1371/journal.pone.0086810 (PMC3922723; doi:10.1371/journal.pone.0086810)
Supplement: Table S1 — Comparison of IFN-γ+ cells population between Pd-sensitization state. Fifteen hours after Pd challenge, SLN cells were isolated and analyzed for IFN-γ production. Each percentages were analyzed by flow cytometry. (DOCX) [file pone.0086810.s004.docx]

Table S1: Comparison of IFN-γ^+^ cell population between Pd-sensitization state.

|  | | | Gated on | | | |
| --- | --- | --- | --- | --- | --- | --- |
|  | Sensitized with | Challenged with | Lymphocytes | T cells | CD4^+^ T cells | CD8^+^ T cells |
| Naïve | None | None | 2.5% | 3.5% | 0.8% | 8.2% |
| Unsensitized | PBS | Pd | 2.8% | 4.7% | 0.1% | 23.6% |
| Sensitized | Pd+LPS | Pd | 13.9% | 20.4% | 0.1% | 41.4% |
